# Do depression literacy, mental illness beliefs and stigma influence mental health help-seeking attitude? A cross-sectional study of secondary school and university students from B40 households in Malaysia

**DOI:** 10.1186/s12889-019-6862-6

**Published:** 2019-06-13

**Authors:** Norhayati Ibrahim, Noh Amit, Suzana Shahar, Lei-Hum Wee, Rozmi Ismail, Rozainee Khairuddin, Ching Sin Siau, Aisyah Mohd Safien

**Affiliations:** 1 Health Psychology Program, Faculty of Health Sciences, Universiti Kebangsaan Malaysia, Kuala Lumpur, Malaysia; 2 Research Centre for Healthy Aging and Wellness, Faculty of Health Sciences, Universiti Kebangsaan Malaysia, Kuala Lumpur, Malaysia; 3 Centre for Community Health, Faculty of Health Sciences, Universiti Kebangsaan Malaysia, Kuala Lumpur, Malaysia; 4 Dietetics Program, Faculty of Health Sciences, Universiti Kebangsaan Malaysia, Kuala Lumpur, Malaysia; 5 Nutrition Sciences Programme, Faculty of Health Sciences, Universiti Kebangsaan Malaysia, Kuala Lumpur, Malaysia; 6 Centre of Human and Societal Well-being, Faculty of Social Sciences and Humanities, Universiti Kebangsaan Malaysia, Bangi, Selangor Malaysia

**Keywords:** Help-seeking, Attitude, Low socioeconomic, Self-stigma

## Abstract

**Background:**

Mental illness rates among young people is high, yet the frequency of help-seeking is low, especially among those from lower socioeconomic backgrounds. Understanding factors influencing help-seeking, such as mental illness beliefs, stigma and literacy among B40 individuals is important, but past studies are sparse. Hence, we aimed to examine the factors associated with mental help-seeking attitude among students from the B40 income bracket. Differences in beliefs toward mental illness, stigma and help-seeking attitudes among university and secondary school students were also investigated.

**Methods:**

University and secondary school students from low-income households (*N* = 202) were involved in this cross-sectional study. Participants completed the Depression Literacy Questionnaire (D-Lit), General Help Seeking Questionnaire (GHSQ), Mental Help Seeking Attitudes Scale (MHSAS), Self-Stigma of Seeking Help Scale (SSOSH), and Beliefs toward Mental Illness (BMI).

**Results:**

Mental help-seeking attitude had a significant relationship with self-stigma on seeking help (r = −.258, *p* < .001), general help-seeking attitude (r = .156, *p* = .027), and age (r = .187, *p* < .001). However, the strongest predictor for mental help-seeking attitude was self-stigma on seeking help (F (2,199) = 8.207, *p* < .001 with R^2^ of .076). University students had better depression literacy and lower levels of self-stigma and negative beliefs toward mental illness compared to secondary school students.

**Conclusion:**

Higher self-stigma and younger age were associated with negative mental help-seeking attitudes among students from low-income households. As self-stigma may be a barrier to actual mental help-seeking, efforts to reduce self-stigma in this population need to be intensified.

## Background

Mental disorders are estimated to affect up to 13.4% of children and adolescents [1]. However, the treatment rate is low. For example, a review on the use of psychiatric treatment found that only around 33% of students with mental health problems were treated [2]. This trend was also observed among young people who showed symptoms of depression and anxiety, of which only 18 to 24% sought professional help [3]. Those who did seek help preferred to obtain assistance from friends and family, rather than a professional [4]. In addition, individuals from lower socioeconomic background have a lower rate of mental health service utilization [5].

Seeking help for mental health issues is the first step toward assessing the mental state, getting the proper diagnosis and subsequently undergoing the intervention and management of mental health by professionals. However, the factors influencing mental help-seeking attitudes need to be explored further. Researchers have identified the barriers and listed several factors; a) technical problems such as financial burden incurred by mental health services [6] and difficult access to the care provider due to transportation or undersized and inadequate resources [7, 8]; b) Personal views such as the lack of perceived need for treatment [9] or perceived ineffectiveness of the therapy [2, 10] and c) stigma. In addition, cultural factors could also influence help-seeking intentions. For example, the practice of making a spiritual diagnosis and treatment among clergy in East Malaysia could delay medical help-seeking [11].

The stigma on mental illness is a concern for helpers, as well as the patients. Individuals with mental disorders are often labeled and stigmatized by society due to their behavior and appearance that are considered to deviate from the norms of society [12]. Stigma refers to an attribute which society considers as undesirable and which leads to the exclusion of an individual from society [13]. Negative stereotypes, prejudice, and discrimination from society could be internalized and generate feelings of incompetence and low self-esteem within an individual, defined as self-stigma [14]. Studies showed that stigma is one of the deterring factors for seeking mental help in various populations, such as among university students [15, 16], faith communities [17], veterans and military personnel [18, 19] and healthcare students and professionals [20]. In addition, mental illness stigma in Asian communities has been associated with the idea of ‘losing face’, especially for the family members of the mentally ill [21].

The mental health literacy of an individual with mental health problems is another factor that could predict his or her attitudes toward seeking help. Mental health literacy is a facet of the health literacy concept, defined as an individual’s knowledge and beliefs regarding mental health which help to aid his or her recognition, management, and prevention of mental disorders [22]. Thus, mental health literacy means understanding the signs and symptoms of psychiatric disorders and the need to refer to a specialist for proper treatment. Many people do not receive accurate information about psychiatric disorders and incorrect or misleading information could deprive them of appropriate medical care and proper support [23]. The inadequacy of an individual’s mental health literacy may lead to an unwillingness to seek help, while the former’s improvement has been shown to increase help-seeking intentions [24]. However, results on mental health literacy and help-seeking intentions are mixed and require further exploration [25].

Lower socioeconomic status is associated with lower education level, poor quality of housing, unemployment, and financial debt, and these factors are linked to the increased prevalence of mental illness [26–28]. Past research has indicated that individuals with lower socioeconomic status reported increased stigma toward mental illness [29], and decreased mental health literacy [29] and mental help-seeking attitude [30]. However, research on mental illness stigma, literacy and help-seeking intentions in conjunction with socioeconomic status is sparse.

The Malaysian government has divided household income into three categories; Top 20% (T20), with a median income above RM13,148 [31], Middle 40% (M40) with income ranging from RM3,860 to RM8,319 and lastly Bottom 40% (B40) with earnings of RM3,900 a month or less [32]. The B40 household group also includes poor households with monthly income lower than the poverty line income. The B40 group is at a higher risk of having poor mental health caused by lower mental health literacy and negative attitudes toward seeking help compared to those with a higher socio-economic status. Studies on mental health stigma, literacy, and attitudes in Malaysia are limited compared to other countries [33]. In addition, there may be cultural variations which necessitate the exploration of these issues in Malaysia [11, 21]. The present exploratory study seeks to examine the factors associated with mental help-seeking attitude among students from the B40 income bracket in Malaysia. In addition, differences in beliefs toward mental illness, mental illness stigma, and mental help-seeking attitudes among university and secondary school students were also investigated.

## Methods

### Study design and participants

This study is a cross-sectional survey carried out among 202 students from low income or B40 households. The sample comprised 127 secondary school students aged 13 to 17 years old and 75 university students aged 18 to 25 years old. Participants from secondary school were residents of a hostel under a non-government organization which houses almost 200 poor students from all over Malaysia. University participants were recruited from one of the universities in the Klang Valley of Malaysia.

### Data collection

Permission was obtained from the relevant authorities of the sites selected for this study. Participants were approached in their classrooms before the lesson commenced. Those who met the inclusion criterion (from the B40 population with household income <RM 3,900 per month) were selected. The participants who did not meet the B40 criterion were excluded from the study. Verbal consent from the caregivers of the younger participants was obtained, whilst the university participants verbally gave their consent. All of the participants were then asked to complete the self-rated questionnaires. Ethical approval was obtained from Universiti Kebangsaan Malaysia Research Ethics Committee.

### Instruments

This study used questionnaires which were translated into Malay for the purpose of this study. A demographic question sheet obtained information on age, gender, and family income.

#### Depression literacy scale [34]

The Depression Literacy Scale or D-Lit was developed by Griffiths et al. [34] to assess mental health literacy specific to depression. It consists of 22 items which can be answered either “true”, “false” or “don’t know” by the participants. Each correct response receives one point and higher scores indicate higher literacy of depression in the participants. It has an internal consistency of Cronbach’s alpha = 0.77 and 0.74 when tested among a Bangladesh population and healthcare professional students in India respectively [35, 36], which reflect a good reliability. For the present study, five items were deleted leaving only 17 items, to achieve the acceptable KR20’s value of Cronbach’s alpha = 0.55. The Cronbach’s alpha value of 0.60 is considered acceptable in social sciences [37].

#### General help seeking questionnaire (GHSQ) [38]

GHSQ has been developed to measure intentions to seek help from different sources and for different problems (personal or emotional problems and suicidal emotion) [38]. The instrument consists of 10 items repeated twice for both problems, with a total of 20 questions, measured with a 7-point Likert scale ranging from “extremely unlikely” to “extremely likely” to find help from different sources such as intimate partner, friend, parent, mental health professional and others. One question is stated as “I would not seek help from anyone” and is reverse scored. Higher scores on this instrument indicate better attitudes on general help-seeking. The reliability of GHSQ when tested as a single instrument is good, with Cronbach’s alpha 0.85 and 0.92 for test-retest reliability, as assessed over a three-week period. GHSQ has been analyzed as two scales, one for each problem-type: suicidal problems (Cronbach’s alpha = 0.83, test-retest reliability assessed over a three-week period = 0.88) and personal-emotional problems (Cronbach’s alpha = 0.70, test-retest reliability assessed over a three-week period = 0.86). Internal consistency for the current study was 0.749.

#### Mental help seeking attitude scale (MHSAS) [39]

This questionnaire is developed by Hammer, Parent, and Spiker [39] to assess attitudes toward seeking help from a mental health professional. It contains nine items and uses a 7-point semantic differential scale. In order to calculate the total score, it is necessary to reverse items 2, 5, 6, 8 and 9. Higher scores indicate more favorable attitudes toward seeking help from mental health professionals. The reliability was good with Cronbach alpha value of 0.92 [39] and for the current study, the value was 0.884.

#### Self-stigma of seeking help scale (SSOSH) [40]

The 10-item SSOSH scale has been developed to assess self-stigma on seeking help regardless of whether an individual has already been diagnosed with a mental illness or not [40]. It consists of a 5-point Likert scale rated from “strongly disagree” to “strongly agree”. The SSOSH obtained good reliability (α = 0.90) and test-retest reliability (α = 0.72) [40–42]. Items 2, 4, 5, 7, and 9 were reverse scored and high scores indicate high levels of self-stigma on seeking help. The scale’s reliability for the current study was Cronbach’s alpha = 0.667.

#### Beliefs toward mental illness (BMI) [43]

BMI has been developed to measure cross-cultural differences in negative beliefs, perceptions and judgments toward mental illness [43]. This scale comprises of 21 questions under three sub-dimensions: gravity, incurability/poor social and interpersonal skills and embarrassment. It uses a 6-point Likert scale and is rated from “completely disagree” to “completely agree”. Higher scores indicate higher levels of negative belief toward mental illness. The reliability of this instrument was good when tested on American, Japanese, and Korean women, with Cronbach’s α value of 0.92 [44] and in the present study was 0.797, indicating good reliability.

### Data analysis

This study used the IBM SPSS Statistics for Windows, version 22.0 (IBM Corp., Armonk, N.Y., USA) to analyze the data. Descriptive analysis was conducted for the description of the mean and standard deviation of all the variables. The Pearson’s correlation test was used to test the correlation between help-seeking domain, self-stigma, negative belief toward mental illness and depression literacy with mental help-seeking. In order to compare all variables based on academic level, t-test analysis was chosen. Lastly, multivariate linear regression was used to test the effect of multiple independent variables (self-help seeking domain, self-stigma, negative belief, and depression literacy) on the dependent variable (mental help-seeking attitude).

## Results

Most of the participants (*N* = 202; *Mean* age = 17.03 ± 3.36) in this study were females (67.8%), and from the second group of B40 household income which is within the range of RM1000-RM3000 (57.9%) (Table 1).

**Table 1:** Demographic profile of the participants (*N* = 202)

Variable	Frequency	Percentage
Gender		
Male	65	32.2
Female	137	67.8
Age		
13–15	90	44.6
16–17	35	17.3
18–20	19	9.4
21 and above	58	28.7
Household income		
< 1000	63	31.2
1000–3000	117	57.9
3001–3860	22	10.9

As shown in Table 2, the mean of depression literacy was 5.01 ± 1.93 out of a total score of 21. For the rest of the scales, out of a total score of 140, the mean for general help-seeking was 85.26 ± 14.17. The score for mental help-seeking was 48.93 ± 9.10 from a total score of 63; self-stigma of seeking help was 26.18 ± 4.86 from a total score of 50; and negative belief toward mental illness was 58.99 ± 11.91 from a total score of 150.

**Table 2:** Mean and standard deviation (SD) of the scales

Domain	Scales	Mean	SD
Depression Literacy	Depression Literacy (D-Lit)	5.01	1.93
General help seeking	General Help Seeking Questionnaire (GHSQ)	85.26	14.17
Mental help seeking	Mental Help Seeking Attitude Scale (MHSAS)	48.93	9.10
Self-stigma of seeking help	Self-Stigma of Seeking Help (SSOSH)	26.18	4.86
Negative belief toward mental illness	Belief toward Mental Illness Scale (BMI)	58.99	11.91

Table 3 demonstrated the t-test analysis on the comparison of mental health literacy with academic level. There were significant differences between the three variables, namely Depression Literacy, Self-Stigma of Seeking Help and Negative Belief Toward Mental Illness when compared according to their academic level whereas help-seeking variables showed no significant difference. University students showed a significantly higher level of depression literacy compared to the secondary school participants. However, an inverse trend was observed for Self-stigma of Seeking Help and Negative Belief toward Mental Illness. All participants showed similar levels in their general help-seeking attitude and mental help-seeking attitude.

**Table 3:** t-test analysis of education level on the variables

Variables	M	F	t	df	p
Depression Literacy					
Secondary school’s student	4.67	.004	−3.22	200	.001
University’s student	5.56				
General Help Seeking Attitude					
Secondary school’s student	84.72	.816	−.71	200	.482
University’s student	86.13				
Mental Help Seeking Attitude					
Secondary school’s student	48.08	7.34	−1.88	186.61	.062
University’s student	50.39				
Self-Stigma of Seeking Help					
Secondary school’s student	26.96	1.080	3.016	200	.003
University’s student	24.87				
Negative Belief toward Mental Illness					
Secondary school’s student	62.37	.318	5.64	200	.000
University’s student	53.27				

As shown in Table 4, Mental Help Seeking Attitude for all participants had a significant positive correlation with General Help Seeking Attitude (r = .156 *p* = .027) and age (r = .187, *p* < 001) as well as a negative correlation with Self-stigma of Seeking Help (r = −.258, *p* < 001). This means higher levels of General Help Seeking Attitude and older age is associated with a higher mental help seeking attitude, while higher levels of self-stigma among the participants is associated with the inhibition of help-seeking behavior. From the educational level perspective, a similar trend could be seen in the attitude of secondary school students. Meanwhile, university students only demonstrated a significant negative correlation between Mental Help Seeking Attitude with Self-stigma of Seeking Help (r = −.305, *p* < 001). From Table 5, we can see that only Self-Stigma of Seeking Help predicted Mental Help Seeking attitude, regardless of the academic level (R^2^ = .066–.093, *p* < 0.05). (Table 5).

**Table 4:** Correlations between Mental Help Seeking Attitude with other variables

Variables/Domains	All participants		Secondary school students		University students	
	p	r	p	r	p	r
Depression Literacy	.372	.063	.307	.091	.487	−.081
General Help Seeking Attitude	.027	.156^a^	.040	.183^a^	.420	.095
Self-Stigma of Seeking Help	.000	−.258^b^	.016	−.213 ^a^	.008	−.305^b^
Negative Belief toward Mental Illness	.166	−.098	.680	−.037	.384	−.102
Age	.008	.187^b^				

^a^Correlation is significant at the 0.05 level (2-tailed)

^b^Correlation is significant at the 0.01 level (2-tailed)

**Table 5:** Multiple Regression to determine the predictors of Mental Help Seeking Attitude

	All participants			Secondary school students			University students		
	B	SE B	β	B	SE B	β	B	SE B	β
General Help Seeking	.064	.045	.100	.106	.064	.147			
Self-Stigma of Seeking Help	−.439	.131	−.235*	−.398	.191	−.185*	−.45	.164	−.305*
R^2^	.076			.066			.093		

*p < 0.05

## Discussion

The present study found that Self-stigma was the strongest predictor for mental health help seeking attitude among teenagers and young adults of low socioeconomic status. These findings may be related to the Modified Labelling Theory developed by Link et al. [45], where people’s self-efficacy is impaired by the label imposed by the public. Internalization of the stigma leads to non-adaptive coping strategies such as avoidance. Self-stigma on seeking help hinders an individual from getting treatment for their problems, either through psychiatric management or counselling therapy. Previous studies [46, 47] reported that those with psychological concerns hide their problems and avoid treatment to limit the harmful consequences they may experience from psychological services. One harmful aftereffect is the sabotaging of their self-esteem. Seeking professional psychological help is perceived as a threat to self-esteem [48], and a sign of weakness and acceptance of failure [40]. Those who consider seeking help for mental health problems for the first time are likely to be labeled as a mentally ill patient, and to be discriminated by society [49]. Scheff’s (1966, cited in Corrigan) [50] Labelling Theory asserted that the label “mentally ill” leads society to treat the labeled individual abnormally and differently. Individuals who get treatment for depression are considered less emotionally consistent, less interesting and with lower self-esteem compared to people who get treatment for physical diseases. An experimental study by Sibicky and Dovidi [51] found that individuals who attended psychological counseling and psychotherapy faced more negative attitudes compared to those who did not undergo such therapy.

In this study, general help-seeking attitude was positively and significantly related to mental help-seeking attitude. Higher readiness and likeliness to get help from different people, professionals and non-professionals, indicates their favorability on seeking help for mental problems. Previous studies, such as in Horwitz [52] showed that people will talk to at least four members of their social network about their personal concerns before seeking professional help from the psychiatrist. Meanwhile, the Research of Consortium Study [53] and Eisenberg et al. [2] reported that the majority of the students who experienced mental health problems get advice and support from non-professionals, such as family members, romantic partner, roommate, or friends. This may indicate that seeking help from social support (peers and family) could serve as a stepping stone to receive appropriate management (mental health professional) in the future.

The present study found that university students who were older had a better mental help-seeking attitude. Other studies also observed that attitudes toward seeking help are better in older adults compared to younger adults [54]. A study on primary and secondary school students in Scotland reported that the participants would delay or avoid disclosing their mental health problems due to their own perceptions of the symptoms as ‘weird’ or ‘rare’ and feared stigmatization from the peers, teachers and parents once they opened up [55]. Strong parental influence among younger adolescents may be a barrier in seeking professional help [56].

For educational level, three variables were found to be significantly different among participants. University participants showed significantly better depression literacy, and lower levels of self-stigma on seeking help and negative belief toward mental illness compared to the secondary school participants. University students may have better knowledge of depression as they are exposed to a broader social network and have more opportunities to learn about mental health. Higher education also means more access to health information and a better understanding of such information to help them utilize it for the benefit of their mental health state. The negative belief and stigma toward mental illness were more likely to occur among those with lower education [57, 58]. It is interesting to note that although educational level influences these variables in the present study, it does not affect the mental help seeking attitude of the participants. This may be partly due to the equal availability of mental health services in both settings (i.e. secondary and tertiary education) in Malaysia.

This study has a few limitations. As this is a cross-sectional study on B40 students from a few educational institutions in the Klang Valley, Malaysia, the generalizability of the results should be made with caution. In addition, comparisons of the findings of this study with other studies should also be interpreted with caution and need to take into account differences in methodology and context. Future studies should consider employing the qualitative method in order to obtain a more in-depth understanding of existing results, especially on the differences found between secondary school and university students. The intention to utilize mental health services could also be influenced by other factors not investigated in this research, such as knowledge about the availability of mental health services and other perceived barriers, and these could be further investigated in future studies. Finally, sociocultural aspects, such as religion and ethnicity, need to be investigated in future studies as help-seeking behavior may be different across cultures and in different countries.

## Conclusion

This study demonstrated that higher self-stigma and younger age were associated with negative mental help-seeking attitudes among students from low-income households. These findings may serve as a guide for the improvement of psycho-education efforts targeting self-stigma in all academic settings, especially among younger students, as they may indirectly enhance the attitude of Malaysians toward help-seeking for mental health problems.

## Abbreviations

ANOVA: Analysis of Variance
B40: Below 20%
BMI: Beliefs toward Mental Illness Scale
D-Lit: Depression Literacy Questionnaire
GHSQ: General Help Seeking Questionnaire
M20: Middle 20%
MHSAS: Mental Help Seeking Attitude Scale
RM: Ringgit Malaysia
SPSS: Statistical Package of Social Sciences
SSOSH: Self-Stigma of Seeking Help Scale
T20: Top 20%
WHO: World Health Organization
